# Predicting potential distributions of dominant species and assessing future afforestation risks in Guangdong limestone regions under climate change

**DOI:** 10.3389/fpls.2026.1870529

**Published:** 2026-07-22

**Authors:** Huisen Zheng, Qinglian Jiang, Jiayi Zhou, Yongbin Wu

**Affiliations:** College of Forestry and Landscape Architecture, South China Agricultural University, Guangzhou, China

**Keywords:** bivariate spatial mapping, centroid migration, climate change, karst afforestation, MaxEnt, northern Guangdong limestone region, species richness

## Abstract

Climate change is reshaping species distributions across karst landscapes, yet large-scale ecological restoration in southern China remains hampered by species and site mismatch, rooted in inadequate macro-scale assessment of climate–vegetation equilibrium. Existing species distribution model (SDM) studies in karst regions are dominated by single-species, locally calibrated analyses. Here we assembled occurrence records spanning the full Chinese range of 12 dominant tree species (1,376 records) and combined them with bioclimatic, soil, and topographic predictors. We built optimized MaxEnt models and projected suitable habitat for the northern Guangdong limestone region under the SSP1-2.6, SSP2-4.5, and SSP5-8.5 scenarios for 2061–2080. We then integrated these projections into a bivariate spatial framework that couples current species richness with net richness change (ΔS) to delineate climate trajectory zones. Models performed well (AUC 0.907–0.984; TSS 0.749–0.933). Annual precipitation (BIO12), precipitation of the driest month (BIO14), and mean temperature of the warmest quarter (BIO10) were the most pervasive drivers across the assemblage. Total suitable area changed by less than 10% across all scenarios. Highly suitable habitat, however, often contracted even where total area held steady, identifying habitat-quality erosion rather than extent loss as the principal threat. Under SSP1-2.6 it shrank for 11 of 12 species, most in *Toona sinensis* (−88%) and *Celtis sinensis* (−85%), while *Sinosideroxylon wightianum* alone gained (up to +208%). Centroids of highly suitable habitat migrated 3.3–90.9 km in varying directions, most erratically under SSP1-2.6. Counterintuitively, the SSP1-2.6 scenario produced the most severe degradation. Its high-risk degradation zone covered 85.2% of the region, against 51.0% under SSP2-4.5 and 54.0% under SSP5-8.5. Critically, areas of highest current species richness coincided spatially with the largest climate-driven degradation risk. Within this high-richness core, the climatically stable zones of the limestone mountains of Yingde City and Yangshan County emerged as the most resilient afforestation targets. These findings provide a spatially explicit, climate-informed framework for tree species selection and afforestation site prioritization in the karst regions of southern China, directly supporting regional ecological restoration planning and long-term forest management under climate change.

## 1 Introduction

Global climate change has profoundly intensified rocky desertification across karst landscapes by altering the hydrological and thermal regimes upon which vegetation depends (Bellard et al., 2012; Thomas et al., 2004), compounding the already severe constraints imposed by natural geological conditions and anthropogenic disturbances (Gao et al., 2021; Zhang et al., 2016). The karst region in southwestern and southern China, centered on Yunnan, Guangxi, and Guizhou provinces, constituting the largest and one of the most contiguous karst landscapes globally and serving as a critical ecological and water security barrier for the upper reaches of the Yangtze and Pearl Rivers (Guo et al., 2013; Jones and White, 2019; Li et al., 2021; Zerga, 2024; Zhang et al., 2016). Endemic plant species in these highly heterogeneous habitats exhibit extreme sensitivity to climate fluctuations, facing elevated extinction risk under ongoing warming (Geekiyanage et al., 2019; Manes et al., 2021; Yan et al., 2024). Critically, warming does not merely threaten these native species *in situ* and it relocates the climatic space that sustains them, shifting rather than simply shrinking their potential range (Feeley et al., 2020; Kelly and Goulden, 2008; Lenoir et al., 2020). On land, however, this relocation is frequently idiosyncratic and lags behind shifting isotherms, because dispersal is physically constrained and human land use impedes tracking (Lenoir et al., 2020), so that the climate a site offers today may no longer sustain the native species growing or planted there within the lifespan of a stand. Systematically assessing the potential impacts of climate change on species distributions is therefore imperative for biodiversity conservation and the formulation of climate adaptation strategies (Hu et al., 2025; Mao et al., 2024; Zhao et al., 2025). To mitigate rocky desertification, large-scale ecological restoration programs, including the Grain for Green Program, have been implemented across southwest China since 1999 (Qiao et al., 2021; Tong et al., 2018). Despite these efforts, afforestation success rates remain limited, primarily due to species and site spatial mismatch: current planning relies excessively on micro-scale physiological assessments while neglecting the macro-scale equilibrium between native vegetation and its broader climatic environment (Li et al., 2025; Trac et al., 2007; Zhang et al., 2022). Resolving this mismatch requires a shift toward macro-scale spatial frameworks capable of capturing species and climate equilibrium relationships.

Species distribution models (SDMs) provide precisely this macro-scale framework, correlating species occurrences with environmental factors to predict potential geographical distributions (Araújo et al., 2019; Elith et al., 2006). The validity of these projections, however, relies heavily on capturing the species’ fundamental ecological niche (Guisan et al., 2017). A common pitfall is niche truncation, which occurs when models are calibrated within a geographic extent too restricted to encompass the full range of environmental conditions the species experiences (Anselmetto et al., 2025; Pang et al., 2022). Such truncation inevitably leads models to overestimate the extent of suitable habitat. Most SDM studies emphasize either broad-scale niche characterization or local-scale application, yet rarely integrate the two. For karst afforestation, a framework that explicitly calibrates the niche across both scales is therefore still lacking (Francisconi et al., 2026). Moreover, current planning frameworks rarely integrate the species-richness baseline with future climate-induced vulnerability (Elith and Leathwick, 2009; Titeux et al., 2017; Wang et al., 2019). Bridging this gap requires scaling up from individual projections to a multi-species framework. By quantifying where ecological value and climate risk intersect, it delivers the community-level spatial guidance needed for multi-species afforestation planning (Meyer and Pie, 2018). To address this gap, our study calibrates models at a broad spatial extent that spans each species’ full Chinese range, capturing the complete climatic niche and the predictions are then clipped to the northern Guangdong limestone region (Chauvier et al., 2022). By moving from a broad scale to a local scale, this projection strategy mitigates niche truncation, thereby offering a scientific basis for forest management and afforestation under climate change and underscoring the value of aligning forestry planning with regional climatic conditions (Araújo and Pearson, 2005; Pearson and Dawson, 2003).

As a vital eastern component of the southern China karst belt, the northern Guangdong limestone area is an ecologically important yet understudied ecosystem. Lying in the upstream catchments that feed the Pearl River Delta, it serves as an ecological barrier and water-source region for the highly urbanized Guangdong–Hong Kong–Macao Greater Bay Area, yet despite severe rocky desertification and intense social-ecological pressures (Liu et al., 2024), restoration research here has lagged behind that in the broader southwestern karst plateau of Guangxi, Guizhou, and Yunnan, leaving the climate-adaptive distributions of its dominant woody species poorly characterized at the regional scale. The present study therefore focuses on 12 dominant woody species: *Zenia insignis* Chun, *Quercus glauca* Thunb., *Choerospondias axillaris* (Roxb.) B. L. Burtt & A. W. Hill, *Triadica rotundifolia* (Hemsl.) Esser, *Lindera communis* Hemsl., *Toona sinensis* (Juss.) Roem., *Sinosideroxylon wightianum* (Hook. & Arn.) Aubrév., *Celtis sinensis* Pers., *Castanea mollissima* Blume, *Distylium myricoides* Hemsl., *Boniodendron minus* (Hemsl.) T. Chen, and *Melia azedarach* L. These species were selected because they are the woody taxa most consistently observed as canopy constituents of the secondary limestone forests across the study area during our field surveys, and because they are the species most commonly used in local afforestation and near-natural restoration practice. Based on an optimized MaxEnt model, our specific objectives were to: (1) identify the key environmental factors driving species distributions, and thus the climatic constraints that should guide species selection for afforestation; (2) project the spatiotemporal shifts and centroid-migration trajectories of potential suitable areas for 2061–2080 under the Shared Socioeconomic Pathway (SSP) scenarios (SSP1-2.6, SSP2-4.5, and SSP5-8.5), thereby identifying the species and locations facing the greatest future climate risk; (3) construct a bivariate spatial map overlaying current species richness with projected net richness change (ΔS) to delineate potential priority zones where ecological value and climate resilience coincide; and (4) integrate these outputs into actionable, spatially explicit guidance for climate-resilient afforestation planning across the karst regions of southern China. We tested two hypotheses: (1) Moisture availability, particularly precipitation, is the primary climatic driver limiting species distributions in this region; (2) The spatial interplay between current species richness and projected net richness change (ΔS) governs afforestation priorities. Under this hypothesis, areas of high current richness but large projected richness loss represent higher-risk afforestation targets than areas of moderate current richness but minimal projected change.

## 2 Material and methods

### 2.1 Study area

The study area is situated in northern Guangdong Province (21°50′–25°31′N, 111°03′–115°22′E), encompassing approximately 54,100 km² across 24 counties and districts in six municipalities, including Qingyuan and Shaoguan (Cui et al., 2015). The region is characterized by a southern subtropical monsoon climate, with a mean annual temperature of approximately 21 °C and annual precipitation of roughly 1,800 mm (Tian et al., 2022). The landscape is dominated by limestone peak forests, depressions, and sinkholes, spanning an elevational gradient from 100 m to 750 m. Severe bedrock exposure, shallow soil profiles, and rapid subterranean water infiltration collectively define the quintessential karst ecological attributes of this region (Ford and Williams, 2007).

### 2.2 Species occurrence data collection

**Figure 1:**
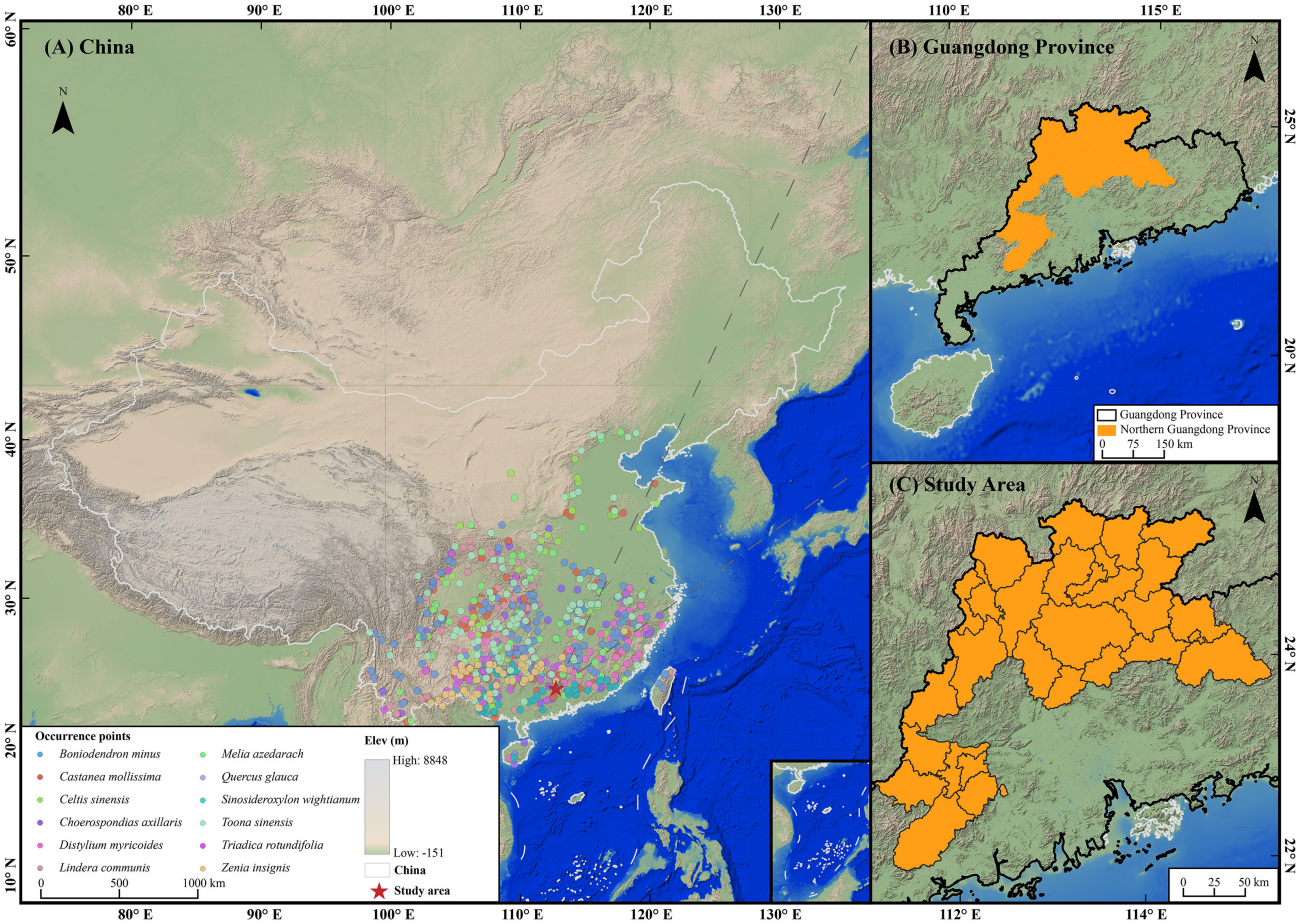
Geographical distribution of occurrence records for 12 dominant tree species in China.

To mitigate niche truncation and better capture each species’ complete climatic niche, occurrence records were compiled from across China. Sources comprised online biodiversity databases, namely the Global Biodiversity Information Facility (GBIF; https://www.gbif.org) and the China Virtual Herbarium (CVH; https://www.cvh.ac.cn/), together with the published literature and field surveys. Only records from 1970 onward were retained to align with the temporal coverage of the environmental variables (Essl et al., 2024). Raw records were screened using the CoordinateCleaner R package (Zizka et al., 2019) to remove common georeferencing errors, such as coordinates in the ocean, at country or province centroids, or at biodiversity-institution localities. Records falling outside each species’ native range or in climatically implausible areas were also removed. To reduce spatial autocorrelation and sampling bias, the ENMTools (Warren et al., 2010) software was employed to spatially thin the data, ensuring that only one occurrence record was retained per 2.5 arc-minutes resolution grid cell (Stark and Fridley, 2022). After screening, 1,376 valid occurrence records were obtained for model calibration (**Figure 1**). These included *Z. insignis* (58), *Q. glauca* (165), *C. axillaris* (155), *T. rotundifolia* (80), *L. communis* (149), *T. sinensis* (131), *S. wightianum* (86), *C. sinensis* (119), *C. mollissima* (114), *D. myricoides* (158), *B. minus* (66), and *M. azedarach* (95).

### 2.3 Environmental variable collection and selection

To adequately capture the relationship between the suitable distributions of species and environmental drivers, climatic, edaphic, and topographic variables were selected for SDM construction (**Supplementary Table 1**). These three categories jointly define the fundamental niches of plants in highly heterogeneous karst landscapes. Climatic variables set the broad-scale thermal and hydrological boundaries of the niche. Edaphic factors govern water retention and nutrient availability, which become primary survival constraints in karst terrain, where soils are shallow and subterranean drainage is rapid (Thurm et al., 2018). Topographic factors, including elevation, slope, and aspect, in turn dictate local microclimates, surface runoff, and solar radiation. These finer-scale gradients shape the establishment and persistence of the dominant woody species across rugged limestone terrain (Ohmura, 2012). Nineteen bioclimatic variables (BIO1–BIO 19) and elevation data for both the current period (1970–2000) and the future 2070s (2061–2080) were acquired from the WorldClim 2.1 database (http://www.worldclim.org; accessed [April 2026]). For future climate projections, we utilized the BCC-CSM2-MR global climate model (GCM) from the Coupled Model Intercomparison Project Phase 6 (CMIP6), which has been demonstrated to perform well for climate studies in China (Xin et al., 2020). Three SSPs were selected: (1) SSP1-2.6, representing a sustainable development pathway with low greenhouse gas emissions; (2) SSP2-4.5, a middle of the road development pathway with intermediate greenhouse gas emissions; and (3) SSP5-8.5, a fossil fueled development pathway with high greenhouse gas emissions. Eight edaphic factors were extracted from the Harmonized World Soil Database (HWSD v1.2; https://gaez.fao.org/pages/hwsd; accessed [April 2026]). Topographic factors, specifically slope and aspect, were derived from the elevation data using the Spatial Analyst tool in ArcGIS 10.8 (Esri, Redlands, CA, USA). A spatial resolution of 2.5 arc-minutes was adopted to balance computational tractability over the broad, full-China calibration extent with sufficient spatial detail for regional projection, and because it is the finest resolution at which all bioclimatic, edaphic, and topographic layers were jointly available. All layers were resampled to this common 2.5 arc-minute grid, masked to the extent of China, and converted to the ASCII (.asc) format required by MaxEnt. To mitigate the risk of model overfitting induced by multicollinearity among environmental variables, a preliminary run of the MaxEnt model was conducted using all initial variables (Brun et al., 2020; Elith and Graham, 2009). Variables with a percent contribution of less than 1%, as evaluated by the jackknife test, were initially excluded. Subsequently, a Pearson correlation analysis was performed in R and visualized using the corrplot package. For highly correlated variable pairs (Pearson’s *|r|* > 0.8), only the variable exhibiting the higher percent contribution was retained (Ge et al., 2026). Finally, the variance inflation factor (VIF) was computed for the retained variables using the usdm package (Naimi et al., 2014), and any variable with VIF > 10 was removed (Guisan et al., 2017). The final sets of screened environmental variables used for model calibration for each tree species are detailed in **Table 1**.

**Table 1:** Environmental variables used in species distribution modeling for the dominant tree species.

Species	Environmental variables
*Zenia insignis*	BIO2; BIO9; BIO15; BIO16; T_CLAY; Elev; Slo; Asp
*Quercus glauca*	BIO1; BIO2; BIO4; BIO12; BIO14; BIO15; T_GRAVEL; Slo; Asp
*Choerospondias axillaris*	BIO2; BIO5; BIO7; BIO8; BIO12; BIO14; T_GRAVEL; T_CLAY; Slo; Asp
*Triadica rotundifolia*	BIO1; BIO3; BIO7; BIO12; BIO15; BIO18; T_USDA_TEX; Slo; Asp
*Lindera communis*	BIO2; BIO6; BIO12; BIO14; T_GRAVEL; T_CACO_3_; T_SILT; Elev; Slo; Asp
*Toona sinensis*	BIO2; BIO3; BIO10; BIO12; BIO15; T_GRAVEL; T_CLAY; T_CACO_3_; Slo; Asp
*Sinosideroxylon wightianum*	BIO2; BIO3; BIO12; BIO17; BIO18; T_GRAVEL; T_USDA_TEX; Elev; asp
*Celtis sinensis*	BIO2; BIO3; BIO8; BIO10; BIO12; BIO15; T_CLAY; T_GRAVEL; T_OC; T_SILT; Slo; Asp; T_CACO_3_
*Castanea mollissima*	BIO2; BIO6; BIO8; BIO14; BIO15; BIO18; T_GRAVEL; T_CACO_3_; Elev; Slo; Asp
*Distylium myricoides*	BIO2; BIO4; BIO5; BIO8; BIO12; BIO14; T_CLAY; Slo; Asp
*Boniodendron minus*	BIO2; BIO3; BIO8; BIO10; BIO15; BIO16; T_GRAVEL; T_SILT; T_USDA_TEX; Asp
*Melia azedarach*	BIO2; BIO3; BIO8; BIO10; BIO12; T_GRAVEL; T_SILT; T_USDA_TEX; Slo; Asp

### 2.4 MaxEnt model construction, optimization and evaluation

To enhance the rigor and reliability of the model predictions, this study utilized the ENMeval package (Muscarella et al., 2014) in R to systematically optimize the regularization multipliers (RM) and feature classes (FC) of the MaxEnt model (Phillips et al., 2006). The RM influences the concentration of the output distribution, while the FC primarily comprises Linear (L), Quadratic (Q), Hinge (H), Product (P), and Threshold (T) features. In this study, the RM was configured from 0.5 to 5.0 (in increments of 0.5, yielding 10 levels), and the FC was set to eight combinations: L, LQ, LH, LQH, QHP, QHPT, LQHP and LQHPT. Consequently, a total of 80 parameter combinations were cross generated for calibration. By evaluating model complexity and the degree of overfitting, the candidate model exhibiting a difference in the sample size corrected Akaike Information Criterion (Δ AICc) of 0 and a lower 10% training omission rate (OR_10_) was selected as the optimal model for each dominant species (Burnham and Anderson, 2002; Merow et al., 2014; Warren and Seifert, 2011). The optimal parameters for the 12 species were determined as follows: *Z. insignis* (RM: 2.0, FC: LQH), *Q. glauca* (RM: 2.5, FC: LQHPT), *C. axillaris* (RM: 2.0, FC: LQHP), *T. rotundifolia* (RM: 1.0, FC: LQHP), *L. communis* (RM: 2.5, FC: QHP), *T. sinensis* (RM: 1.5, FC: LQP), *S. wightianum* (RM: 0.5, FC: L), *C. sinensis* (RM: 2.5, FC: LQH), *C. mollissima* (RM: 2.5, FC: LQH), *D. myricoides* (RM: 1.0, FC: LH), *B. minus* (RM: 0.5, FC: LQ), and *M. azedarach* (RM: 1, FC: LQHPT). Prior to formal model construction, the occurrence data for each species and the screened environmental layers were imported into MaxEnt version 3.4.1, applying the aforementioned optimized parameters. The software was configured to randomly partition 75% of the occurrence records for model training, allocating the remaining 25% for testing. The maximum number of iterations was set to 1,000, with 10 bootstrap replicates. To assess the relative influence of environmental drivers, we evaluated two complementary metrics provided by MaxEnt, including percent contribution and permutation importance. Percent contribution reflects the heuristic accumulation of each variable’s contribution to model gain during the iterative training process, and therefore depends on the path by which the model is fitted. Permutation importance, by contrast, is computed after training by randomly permuting the values of each variable among the presence and background points and measuring the resulting decrease in training area under the receiver operating characteristic curve (AUC; Swets, 1988); it thus reflects the model’s reliance on that variable and is less sensitive to the training path and to correlations among predictors. Both metrics are reported for each species (**Table 2**). Variables with a percent contribution exceeding 10% were regarded as the primary environmental factors shaping the regional distribution of the dominant tree species (Jarnevich et al., 2015). Predictive performance was assessed for each species using two complementary metrics. The AUC, a threshold-independent measure of discrimination, was obtained from the MaxEnt output, with values closer to 1 indicating better performance. To complement AUC with a threshold-dependent measure, we also computed the True Skill Statistic (TSS = sensitivity + specificity − 1; Allouche et al., 2006). For each of the 10 bootstrap replicates, TSS was calculated in R (dismo package; Hijmans et al., 2017) from the withheld test localities of that replicate and a fixed set of 10,000 random background points, and was evaluated at the threshold that maximized TSS; the per-species values reported are the means across the 10 replicates.

**Table 2:** Dominant environmental variables and their percent contributions for the 12 tree species.

Species	Dominant environmental variables	Percent of contribution (%)	Permutation importance (%)
*Zenia insignis*	BIO16	74.5	15.7
*Quercus glauca*	BIO12	53.5	28.3
	BIO1	15.2	24.6
	BIO4	11.1	14.9
	BIO2	10.1	6.7
*Choerospondias axillaris*	BIO14	49	1.3
	BIO12	33	4.5
*Triadica rotundifolia*	BIO12	59.5	18.6
*Lindera communis*	BIO12	36.8	2.1
	BIO14	26.1	1
	BIO6	18.1	71.4
*Toona sinensis*	BIO12	68.7	49.5
	BIO10	10	18
*Sinosideroxylon wightianum*	BIO17	46.9	5.9
	BIO18	12.9	16.7
	BIO3	11.7	16
	BIO12	11.4	4
*Celtis sinensis*	BIO12	65.6	10.3
	BIO10	16	56.2
*Castanea mollissima*	BIO14	58	4.2
*Distylium myricoides*	BIO14	73.9	17.9
	BIO12	10	15.6
*Boniodendron minus*	T_USDA_TEX	34.6	4.2
	BIO2	26.3	22.8
	BIO10	15	31.4
*Melia azedarach*	BIO12	70.9	5.5
	BIO10	11.1	41.6

### 2.5 Dynamic changes and migration trends of suitable habitats

The averaged logistic output of the MaxEnt models, representing habitat suitability indices (*P*) ranging from 0 to 1, was imported into ArcGIS. The Reclassify tool was employed to categorize the suitability into four discrete levels: unsuitable habitat (0 ≤ *P* < 0.2), poorly suitable habitat (0.2 ≤*P* < 0.4), moderately suitable habitat (0.4 ≤ *P* < 0.6), and highly suitable habitat (*P* ≥ 0.6) (Liu et al., 2026b; Xiao et al., 2022a).Subsequently, the Extract by Mask tool was utilized to delineate the potential distribution maps specifically for the northern Guangdong limestone study area. Finally, raster calculations and zonal statistics were conducted to analyze the spatiotemporal shifts in the potential distribution ranges and suitable areas for each tree species within this limestone region under future climate scenarios. Besides, to assess the temporal dynamics of the spatial centroids of highly suitable habitats across different periods, we first applied a binarization process to the habitat suitability layers. Highly suitable habitats were defined as highly suitable habitats and assigned a value of 1, yielding a binary map for each species. Subsequently, the SDMtoolbox 2.4 extension in ArcGIS 10.8 was employed to calculate the spatial centroids of these highly suitable habitats under both current and future climate scenarios (Brown et al., 2017). This enabled us to quantitatively track and map the spatiotemporal distribution patterns and centroid migration trajectories of the potential suitable habitats for each dominant tree species.

### 2.6 Risk assessment for afforestation

Binary habitat suitability layers for all 12 species were spatially stacked to generate a current species richness layer, where each pixel value represents the number of species for which that location is classified as suitable (Calabrese et al., 2014). It should be noted that stacking binary SDMs is known to overestimate absolute richness values (Pineda and Lobo, 2012). Therefore, this layer is interpreted in a relative sense as a baseline for quantifying directional change. Species richness change maps were computed for each SSP scenario by pixel wise subtraction, quantifying the magnitude and direction of climate-driven niche displacement. The calculation formula is as follows:


$$\Delta S=S_{future}-S_{current}$$

where *S*_future and *S*_current are the pixel-level species richness under future and current climate, respectively, and ΔS is their net change.

A bivariate spatial matrix was constructed integrating current richness (horizontal axis) with net richness change (vertical axis). Horizontal axis breakpoints were initially determined using Fisher’s natural breaks algorithm and subsequently rounded to the nearest integer, yielding three classes: low (0–2 species), moderate (3–6 species), and high (7–12 species). Vertical axis thresholds were fixed at −1 and +1 across all scenarios, isolating pixels of net stability (Δ*S* = 0) as a discrete category independent of scenario specific data distributions. This yielded three climate trajectory zones: high-risk degradation zones (Δ*S* ≤ −1), climatically stable zones (Δ*S* = 0), and climatically gaining zones (Δ*S* ≥ 1). It should be noted that ΔS = 0 reflects net richness stability rather than compositional invariance, as individual species gains and losses may cancel at the pixel level; these zones are interpreted as areas of high climate buffering capacity analogous in function to microrefugia (Keppel et al., 2012; Morelli et al., 2016). Similarly, climatically gaining areas reflect projected suitability gains and do not imply successful colonization, given dispersal constraints inherent to fragmented karst landscapes.

To enable direct cross scenario visual comparison, a unified color classification scheme was applied across all bivariate maps, with the vertical axis spanning the study area ΔS range observed across all scenarios (−9 to +4). Bivariate maps were produced in R using the biscale and ggplot2 packages.

## 3 Results

### 3.1 Model accuracy evaluation

**Figure 2:**
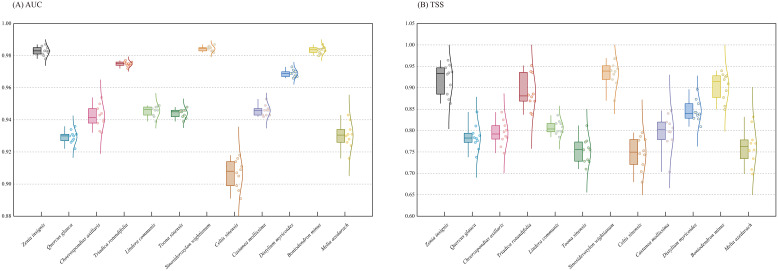
Predictive performance of the optimized MaxEnt models for the 12 dominant tree species, evaluated by **(A)** the area under the receiver operating characteristic curve (AUC) and **(B)** the True Skill Statistic (TSS), each across 10 bootstrap replicates. For every species, the box shows the median and interquartile range, whiskers the data range, jittered points the 10 individual replicate values, and the adjacent curve the kernel density of the distribution. AUC was obtained from the MaxEnt output; TSS was computed from the withheld test localities and a fixed background sample, evaluated at the threshold maximizing TSS.

All 12 optimized MaxEnt models performed well across the evaluation metrics. Mean AUC ranged from 0.907 (*C. sinensis*) to 0.984 (*S. wightianum* and *B. minus*), with every value exceeding 0.900 (**Figure 2**). The True Skill Statistic (TSS) ranged from 0.749 (*C. sinensis*) to 0.933 (*S. wightianum*). The two metrics agreed at the extremes, ranking *C. sinensis* lowest and *S. wightianum* highest in discriminative ability. These results indicate high predictive accuracy and support using the models to project potential distributions under current and future climate conditions.

### 3.2 Major environmental variables affecting the distribution of 12 dominant tree species

The primary environmental drivers identified across the 12 species included annual precipitation (BIO12), precipitation of the driest month (BIO14), precipitation of the wettest quarter (BIO16), precipitation of the driest quarter (BIO17), precipitation of the warmest quarter (BIO18), annual mean temperature (BIO1), mean diurnal range (BIO2), isothermality (BIO3), temperature seasonality (BIO4), mean temperature of the warmest quarter (BIO10), and topsoil texture (T_USDA_TEX) (**Table 2**).

Precipitation variables mainly governed the suitable areas of *Z. insignis*, *T. rotundifolia*, *C. axillaris*, *C. mollissima*, *D. myricoides*, and *S. wightianum*. Precipitation of the wettest quarter (BIO16) was the dominant factor for *Z. insignis*, with a contribution of 74.5%. Annual precipitation (BIO12) was the single leading variable for *T. rotundifolia*, with a contribution of 59.5%, and also contributed 33.0% and 10.0% to *C. axillaris* and *D. myricoides*, respectively. Precipitation of the driest month (BIO14) was the single leading variable for *C. mollissima* and *D. myricoides*, with contributions of 58.0% and 73.9%, and contributed 49.0% to *C. axillaris*. For *S. wightianum*, precipitation of the driest quarter (BIO17) was the leading variable, with a contribution of 46.9%, while precipitation of the warmest quarter (BIO18), isothermality (BIO3), and annual precipitation (BIO12) also exceeded the 10% threshold, contributing 12.9%, 11.7%, and 11.4%, respectively.

Five species had distributions shaped synergistically by precipitation and temperature including *Q. glauca*, *T. sinensis*, *C. sinensis*, *M. azedarach*, and *L. communis*. Annual precipitation (BIO12) was the leading variable for all five, contributing 53.5%, 68.7%, 65.6%, 70.9%, and 36.8%, respectively. Among temperature variables, mean temperature of the warmest quarter (BIO10) exceeded 10% for *T. sinensis*, *C. sinensis*, and *M. azedarach*, with contributions of 10.0%, 16.0%, and 11.1%, respectively. For *Q. glauca*, annual mean temperature (BIO1), temperature seasonality (BIO4), and mean diurnal range (BIO2) also exceeded the threshold, contributing 15.2%, 11.1%, and 10.1%, respectively. For *L. communis*, annual precipitation (BIO12), precipitation of the driest month (BIO14), and minimum temperature of the coldest month (BIO6) were all primary variables, contributing 36.8%, 26.1%, and 18.1%, respectively. The distribution of *B. minus* was distinctive in being led by an edaphic factor. Topsoil texture (T_USDA_TEX) contributed 34.6%, followed by mean diurnal range (BIO2) and mean temperature of the warmest quarter (BIO10), contributing 26.3% and 15.0%, respectively. This was the only species for which a soil variable, rather than a climatic variable, was the leading predictor.

Among the primary variables, annual precipitation (BIO12) was the most pervasive, contributing more than 10% in nine species, with contributions ranging from 10.0% to 70.9%. Precipitation of the driest month (BIO14) contributed more than 10% in four species, ranging from 26.1% to 73.9%. Mean temperature of the warmest quarter (BIO10) was the most frequent temperature variable, contributing more than 10% in four species, ranging from 10.0% to 16.0%. Overall, precipitation variables collectively exerted far greater influence than temperature variables, indicating that water availability, particularly annual precipitation (BIO12), was the primary climatic factor determining the potential distribution of the dominant woody species in the northern Guangdong limestone region.

Furthermore, for most species, the percentage contribution aligns with permutation importance, but discrepancies exist for a few species. For example, BIO6 has a higher permutation importance in *L. communis*, while BIO10 has a higher permutation importance in *C. sinensis*, reflecting the degree of dependence between the model and the climate predictor variables.

### 3.3 Response curves of key variables

**Figure 3:**
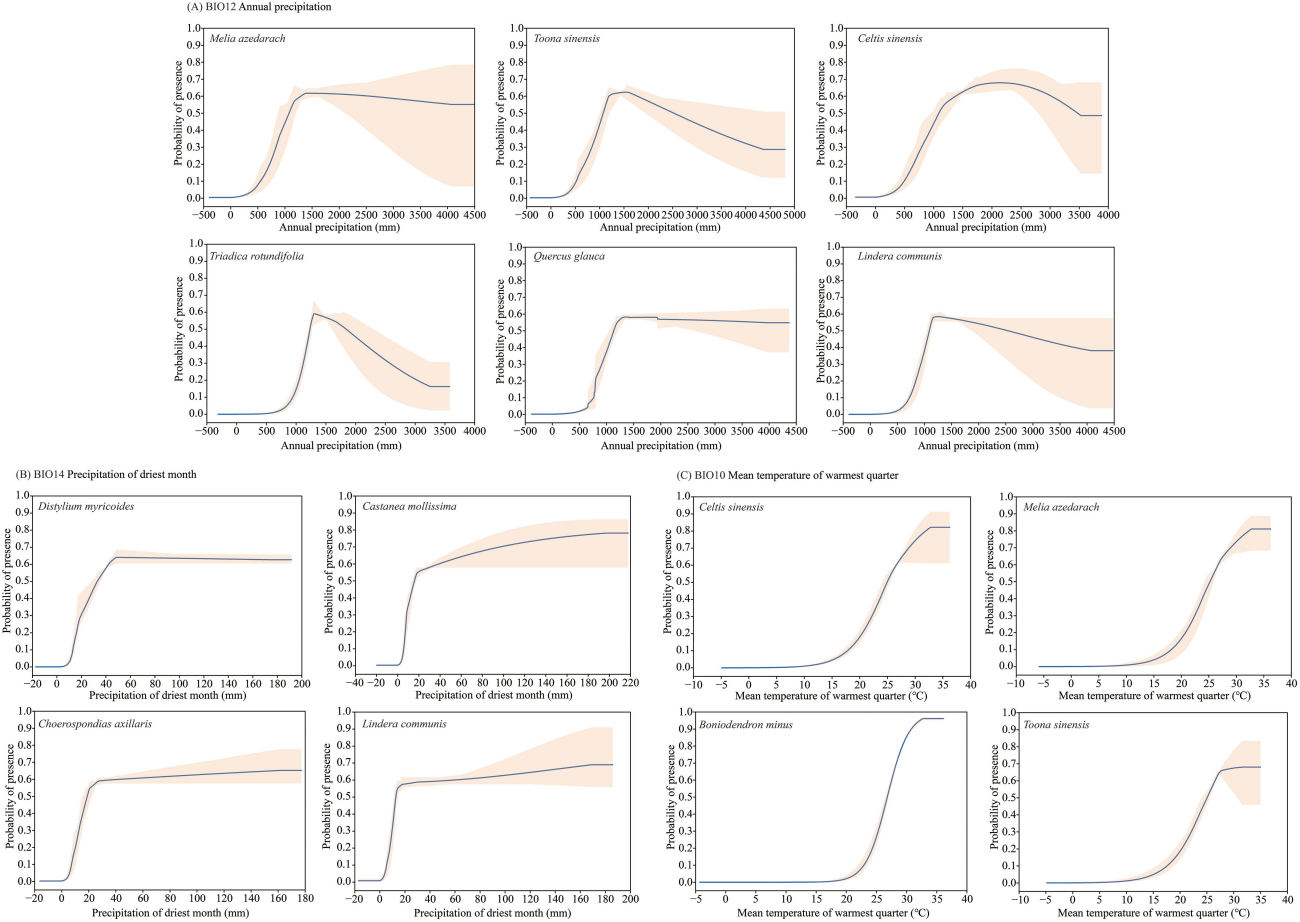
Response curves of the three most influential climatic variables for the dominant tree species, derived from the optimized MaxEnt models and averaged across 10 bootstrap replicates. **(a)** Annual precipitation (BIO12) for the six species for which it was the leading variable; **(b)** precipitation of the driest month (BIO14) for the four species in which it contributed more than 10%; **(c)** mean temperature of the warmest quarter (BIO10) for the four species in which it contributed more than 10%. The y-axis is the logistic habitat suitability index.

To interpret how the leading variables shape habitat suitability, we examined their response curves (**Figure 3**). The response curves for annual precipitation (BIO12), the most pervasive driver, further clarified its influence on habitat suitability in the six species for which it was the leading variable (**Figure 3A**). For all six, suitability rose steeply with annual precipitation at the low end and approached zero below approximately 1,000 mm. This pattern indicates that insufficient annual precipitation is a key constraint on their distribution. Suitability peaked within roughly 1,200–2,150 mm and then plateaued or declined gradually, producing unimodal response curves. The optimal annual precipitation differed among species. It was lowest for *L. communis* (~1,242 mm) and *T. rotundifolia* (~1,305 mm), intermediate for *Q. glauca* (~1,402 mm), *M. azedarach* (~1,410 mm), and *T. sinensis* (~1,553 mm), and highest for *C. sinensis* (~2,142 mm). There are also significant differences in the tolerance ranges among different species. *T. rotundifolia* had the narrowest suitable range, with high suitability confined to ~1,235–1,842 mm, indicating a comparatively narrow precipitation niche. By contrast, *M. azedarach* and *Q. glauca* retained moderate suitability across a much wider range (up to ~4,400 mm). These response shapes indicate that the six species are broadly adapted to humid conditions but are filtered out where annual precipitation falls below roughly 1,000 mm.

The response curves for precipitation of the driest month (BIO14) were examined for the four species for which it was a leading variable (**Figure 3B**). In all four, suitability was near zero when driest-month precipitation approached zero, again indicating that dry-season water shortage strongly limits their distribution. The curve shapes, however, differed. For *C. mollissima*, *C. axillaris*, and *L. communis*, suitability rose monotonically with driest-month precipitation and remained highest at the upper end of the observed range (~160–200 mm). These three species therefore appear favored by progressively wetter dry seasons. In contrast, *D. myricoides* showed a unimodal response, with suitability peaking at a relatively low driest-month precipitation (~49 mm) and declining gradually thereafter. This shape indicates tolerance of, or even preference for, drier conditions in the driest month. *C. mollissima* reached the highest peak suitability (~0.78), consistent with BIO14 being its single dominant driver. Together, these curves indicate that all four species require some dry-season precipitation but diverge in whether higher dry-season rainfall continues to raise suitability or becomes less favorable.

The response curves for mean temperature of the warmest quarter (BIO10) were examined for the four species for which it exceeded the 10% threshold (**Figure 3C**). For all four (*C. sinensis*, *B. minus*, *M. azedarach*, and *T. sinensis*), suitability was near zero below approximately 25 °C. It then rose monotonically with warm-season temperature, remaining highest at the upper end of the observed range (~32–33 °C). This consistent pattern indicates that adequate warm-season heat accumulation promotes habitat suitability for these species, with low summer temperatures acting as a limiting factor. *B. minus* showed the strongest response, reaching a peak suitability of ~0.96. For *C. sinensis*, *M. azedarach*, and *T. sinensis*, by contrast, BIO10 acted as a secondary thermal driver alongside their dominant precipitation variable (BIO12). Overall, the BIO10 response curves indicate that warm-season heat availability, rather than heat stress, shapes the suitability of these species across the temperature range they currently experience.

The response curve for topsoil texture (T_USDA_TEX) showed a clear monotonic decline in suitability from coarse to fine soil textures (**Supplementary Figure 1A**). Suitability was highest on coarse-textured (sandy) soils (~0.76) and decreased steadily as texture became finer, falling below 0.5 at intermediate textures and approaching zero on the finest, clay-rich soils. This indicates a strong preference of *B. minus* for coarse, well-drained substrates, consistent with the shallow, rapidly draining soils characteristic of karst terrain, and provides a clear ecological basis for the unusually high contribution of soil texture for this species.

The response curve for precipitation of the wettest quarter (BIO16), which overwhelmingly governed Z. insignis (74.5%), was unimodal (**Supplementary Figure 1B**). Suitability peaked at approximately 796 mm and declined at both lower and higher values, with high suitability (≥0.5) confined to roughly 690–1,200 mm. This indicates that Z. insignis is favored by a moderate wet-season precipitation regime and is less suited to either drier or excessively wet conditions, defining a comparatively narrow precipitation optimum for this species.

### 3.4 Distribution of 12 dominant tree species under current climate conditions

**Figure 4:**
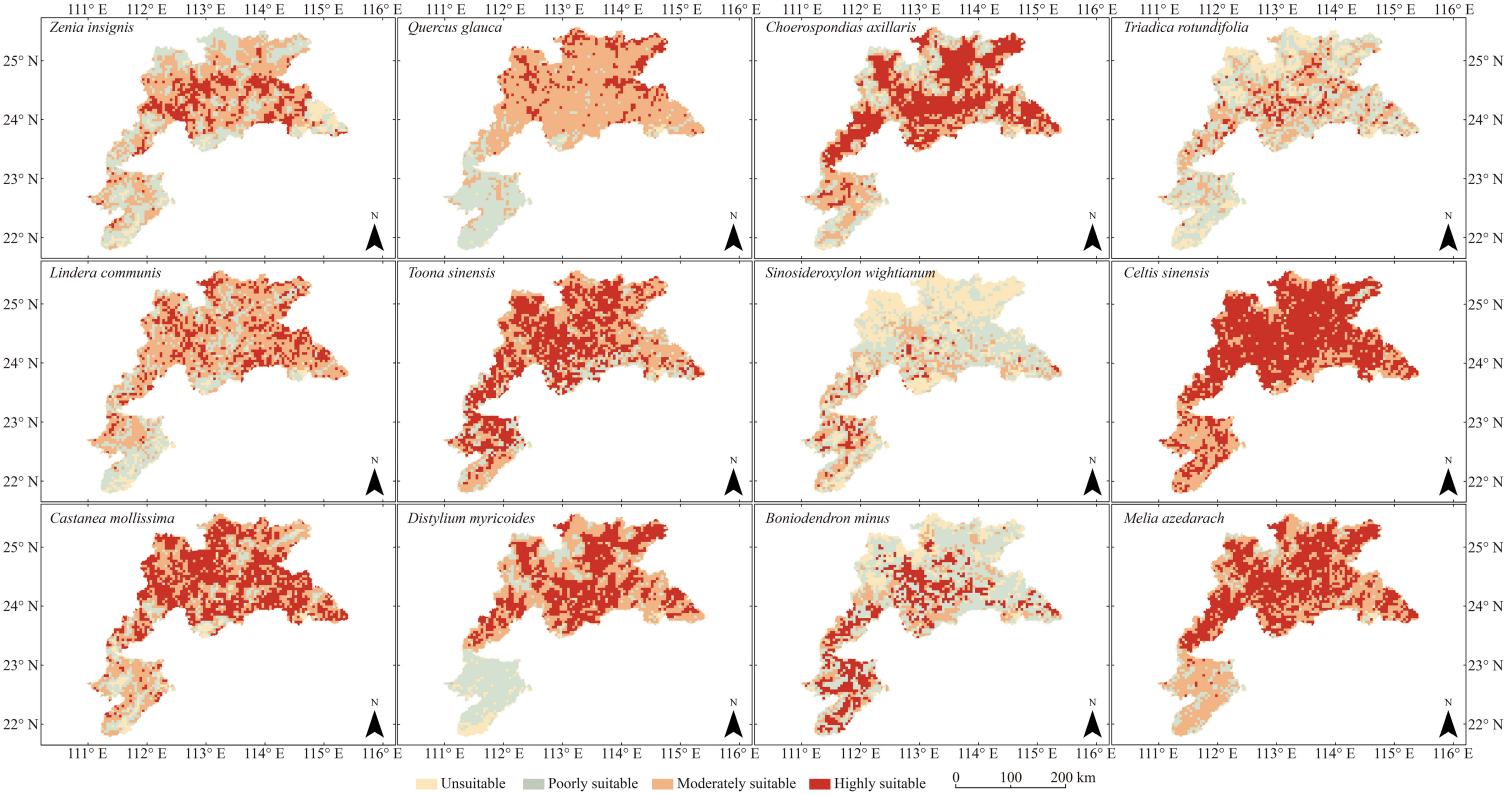
Potential geographical distribution of 12 dominant tree species in the northern Guangdong limestone region under current climatic conditions.

Under current climate conditions (**Figure 4**), the extent of highly suitable habitat varied markedly among the 12 dominant tree species (**Table 3**). *C. sinensis* had the most extensive highly suitable habitat, covering 3.70 × 10^4^ km² (67.8% of the study area), with high suitability concentrated in Yingde City, Huaiji County, and Yangshan County. *M. azedarach*, *T. sinensis*, *C. axillaris*, and *C. mollissima* followed, each with highly suitable areas exceeding 2.3 × 10^4^ km² (42.6–45.7%) and likewise centered on the same core area. For these species, suitable habitat (moderately plus highly suitable) exceeded 4.2 × 10^4^ km², indicating broad regional suitability.

**Table 3:** Suitable habitat areas of the 12 dominant tree species under current climate conditions (×10^4^ km²).

Species	Unsuitable	Low	Moderate	High	High (%)
*C. sinensis*	0.01	0.1	1.65	3.7	67.8
*M. azedarach*	0	0.54	2.42	2.49	45.7
*T. sinensis*	0.03	0.78	2.16	2.48	45.5
*C. mollissima*	0.2	0.72	2.22	2.32	42.6
*C. axillaris*	0.17	1.07	1.84	2.38	43.6
*D. myricoides*	0.2	1.27	2.06	1.92	35.1
*B. minus*	0.73	2.48	1.01	1.23	22.5
*L. communis*	0.17	1.23	2.92	1.13	20.7
*Z. insignis*	0.26	1.75	2.53	0.92	16.9
*Q. glauca*	0.02	1.12	3.64	0.68	12.4
*T. rotundifolia*	0.99	2.3	1.75	0.42	7.6
*S. wightianum*	1.87	2.17	1.15	0.26	4.7

High (%) = highly suitable area as a percentage of the total study area. Species are ordered by highly suitable area.

A second group, comprising *D. myricoides*, *B. minus*, *L. communis*, and *Z. insignis*, was characterized by extensive moderately suitable habitat but more limited highly suitable habitat (1.92, 1.23, 1.13, and 0.92 × 10^4^ km², respectively). Their highly suitable patches were also concentrated in Yingde City, Huaiji County, and Yangshan County. *Q. glauca* showed the most pronounced version of this pattern, with the largest moderately suitable area (3.64 × 10^4^ km², 66.8%) but one of the smallest highly suitable areas (0.68 × 10^4^ km², 12.4%). Unlike the other species, its highly suitable patches were confined to Nanxiong City, Yangshan County, and Lianzhou City, indicating broad but generally low-to-moderate suitability across the region.

In contrast, *S. wightianum* and *T. rotundifolia* were the most spatially restricted, with highly suitable areas of only 0.26 and 0.42 × 10^4^ km² (4.7% and 7.6%) and large unsuitable extents (1.87 and 0.99 × 10^4^ km²). For both species, suitable habitat was confined entirely to Yingde City. Overall, the highly suitable habitat of most species converged on Yingde, Huaiji, and Yangshan. Within this core, *C. sinensis*, *M. azedarach*, and *T. sinensis* were both the most widely and the most highly suitable, whereas *S. wightianum* and *T. rotundifolia* occupied the smallest suitable areas.

### 3.5 Distribution of 12 dominant tree species under future climate change

**Figure 5:**
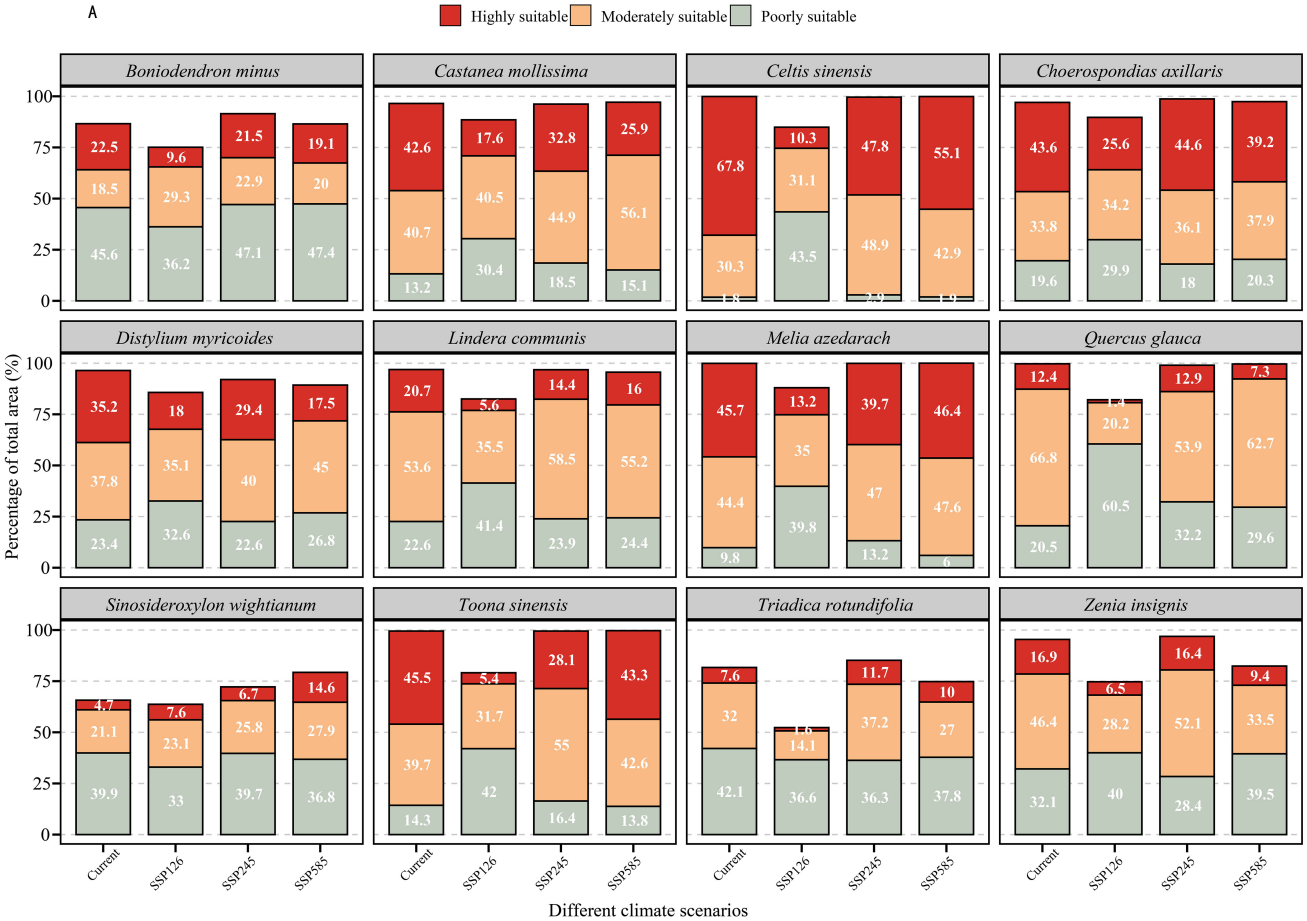
Proportions of different habitat suitability classes for 12 dominant tree species under current and future climate scenarios.

**Figure 6:**
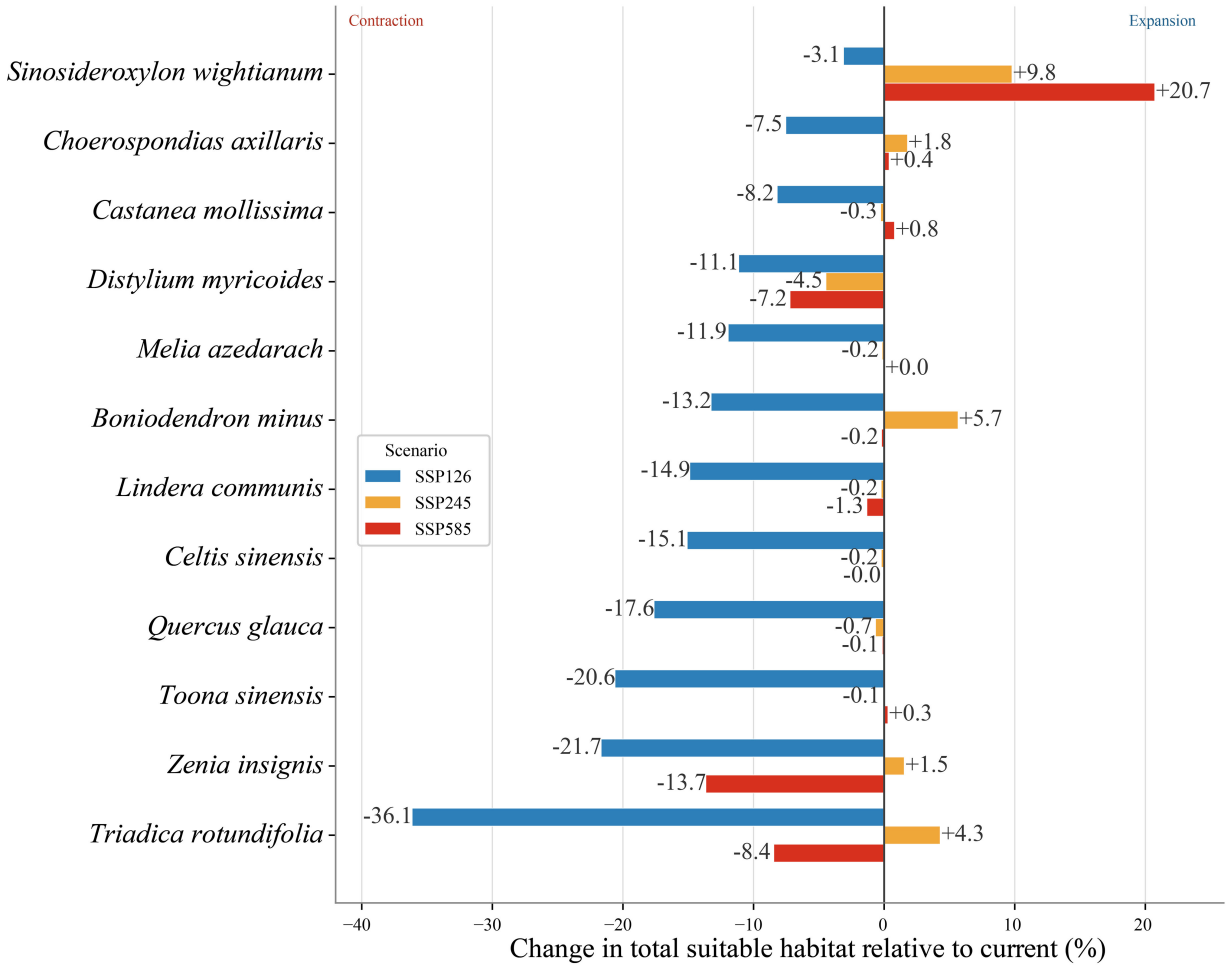
Percentage changes in total suitable habitat area for 12 dominant tree species under future climate scenarios.

The following results report changes in highly suitable habitat (× 10^4^ km²) for each scenario relative to the current baseline (**Supplementary Figures 2**-**4**, **Figures 5**, **6**).

Under SSP1-2.6, highly suitable habitat contracted for 11 of the 12 species. The largest losses occurred in *C. sinensis* (3.70 to 0.56 × 10^4^ km², −85%), *T. sinensis* (2.48 to 0.29 × 10^4^ km², −88%), and *M. azedarach* (2.49 to 0.72 × 10^4^ km², −71%). *C. mollissima*, *C. axillaris*, and *D. myricoides* each lost about 1.0 × 10^4^ km² (−40% to −60%). Only *S. wightianum* gained (0.26 to 0.41 × 10^4^ km², +62%). For several species, total suitable area changed only modestly while highly suitable habitat declined sharply.

Under SSP2-4.5, contraction was less severe. Highly suitable habitat still declined for most species, but by smaller margins (*C. sinensis* 3.70 to 2.61 × 10^4^ km², −29%; *T. sinensis* 2.48 to 1.53 × 10^4^ km², −38%). A few species gained slightly, including *C. axillaris* (+2%), *Q. glauca* (+4%), and *T. rotundifolia* (0.42 to 0.64 × 10^4^ km², +52%). *S. wightianum* remained well above baseline (0.26 to 0.36 × 10^4^ km², +38%).

Under SSP5-8.5, responses were more divergent. *S. wightianum* reached its largest highly suitable area (0.26 to 0.79 × 10^4^ km², +208%), *T. rotundifolia* increased (0.42 to 0.55 × 10^4^ km², +31%), and *M. azedarach* was essentially stable (2.49 to 2.53 × 10^4^ km², +2%). Others continued to lose habitat, including *D. myricoides* (1.92 to 0.96 × 10^4^ km², −50%), *C. mollissima* (2.32 to 1.41 × 10^4^ km², −39%), and *C. sinensis* (3.70 to 3.00 × 10^4^ km², −19%).

Across all scenarios, changes in total suitable area were generally far smaller than changes in highly suitable habitat, and sometimes ran in the opposite direction. In several species, total suitable area remained stable or even expanded while highly suitable habitat contracted. Under SSP5-8.5, for example, *T. sinensis* showed a slight increase in total suitable area but a decline in highly suitable habitat. *C. sinensis*, in turn, retained a large total suitable area while losing highly suitable habitat in every scenario. *S. wightianum* was the only species to expand its highly suitable habitat under all three scenarios, whereas *C. sinensis*, *T. sinensis*, *C. mollissima*, and *D. myricoides* contracted persistently or recurrently.

### 3.6 Centroid migration of highly suitable habitats for dominant tree species under future climate scenarios

**Figure 7:**
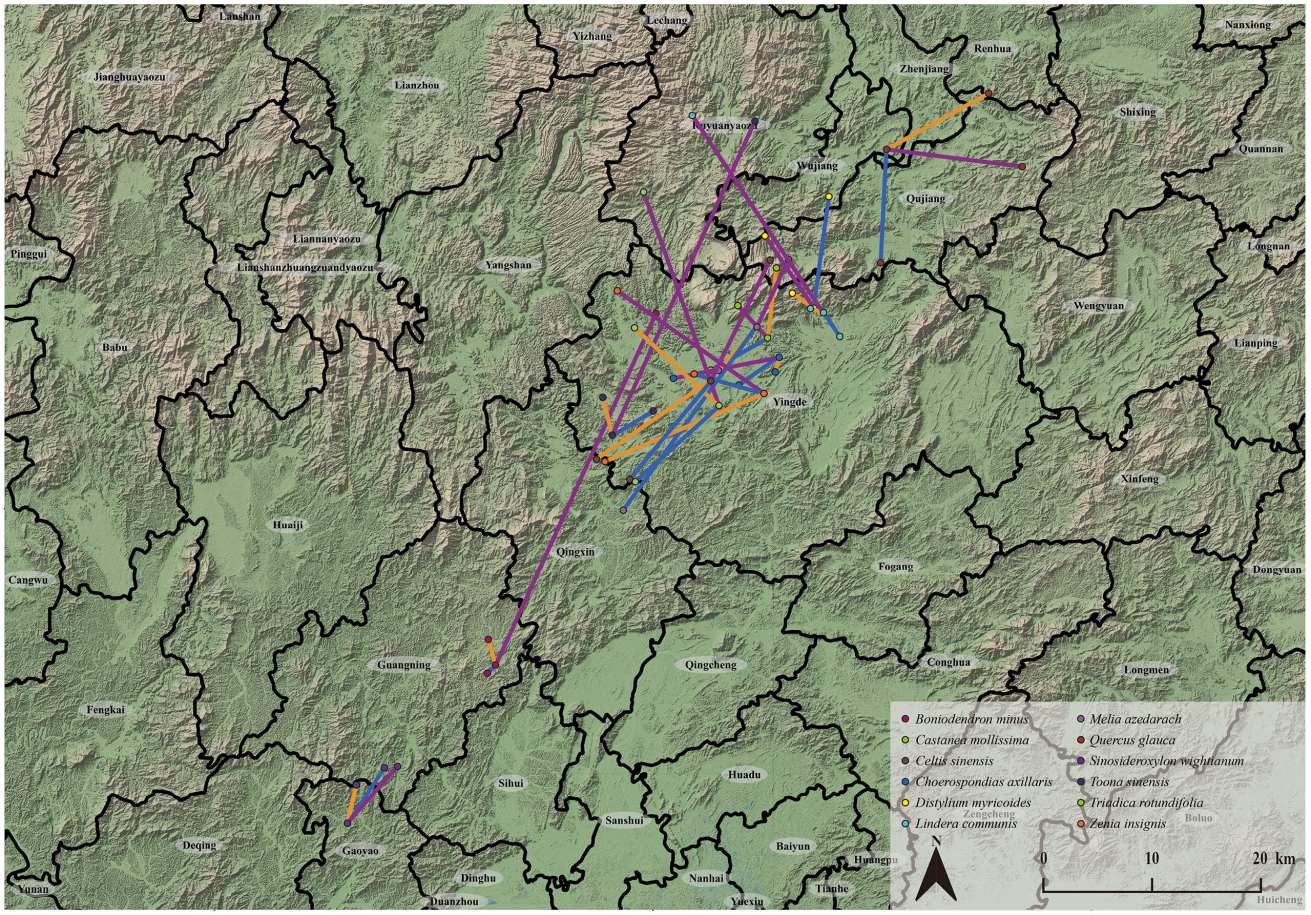
Centroid shifts of highly suitable habitat for 12 dominant tree species under three future climate scenarios (SSP1-2.6, SSP2-4.5, and SSP5-8.5). Colored dots indicate the current centroid location of each species; colored lines radiating from each dot represent the projected centroid displacement under each of the three scenarios, with line color denoting the corresponding SSP pathway. Species are: *Boniodendron minus, Castanea mollissima, Celtis sinensis, Choerospondias axillaris, Distylium myricoides, Lindera communis, Melia azedarach, Quercus glauca, Sinosideroxylon wightianum, Toona sinensis, Triadica rotundifolia,* and *Zenia insignis*.

As shown in **Figure 7**, the centroid trajectories and migration distances of highly suitable habitat differed markedly among the 12 dominant tree species under the three future scenarios. Migration distances ranged from 3.3 km (*L. communis*, SSP2-4.5) to 90.9 km (*B. minus*, SSP1-2.6), and centroids shifted in all directions (northeast, northwest, southeast, southwest) (**Supplementary Table 2**). The displacement was modest for most species, although several underwent unusually large shifts under SSP1-2.6.

Four species migrated along a relatively stable bearing across scenarios. The centroid of *S. wightianum* shifted consistently northeastward (9.1–18.3 km), and that of *C. axillaris* consistently southwestward (3.6–27.3 km). *M. azedarach* and *C. sinensis* migrated predominantly southwestward, holding this bearing under SSP2-4.5 and SSP5-8.5 (reaching 54.2 km and 30.5 km, respectively, under SSP5-8.5) and departing from it only under SSP1-2.6.

The remaining species showed greater directional uncertainty. The centroid of *Q. glauca* migrated southeastward under SSP1-2.6 (34.6 km), northeastward under SSP2-4.5 (28.9 km), and southwestward under SSP5-8.5 (26.0 km). *B. minus* shifted northeastward under SSP1-2.6 (90.9 km), northwestward under SSP2-4.5 (6.3 km), and southwestward under SSP5-8.5 (2.8 km). *C. mollissima*, *T. rotundifolia*, *L. communis*, and *Z. insignis* also reversed direction among scenarios, whereas *D. myricoides* and *T. sinensis* migrated broadly northward but with shifting bearings.

The spatial redistribution of highly suitable habitat was most pronounced and most erratic under SSP1-2.6. Several species underwent their largest centroid shifts under this scenario (*B. minus* 90.9 km, *T. sinensis* 80.8 km, *L. communis* 56.3 km, *T. rotundifolia* 52.9 km), frequently in directions opposite to those under SSP2-4.5 and SSP5-8.5. Under the latter two scenarios, migration distances were generally smaller and directions more consistent. Overall, despite this variation, absolute migration distances remained limited for most species, indicating spatially constrained rather than large-scale displacement of highly suitable habitat.

### 3.7 Priority planting and tending areas

**Figure 8:**
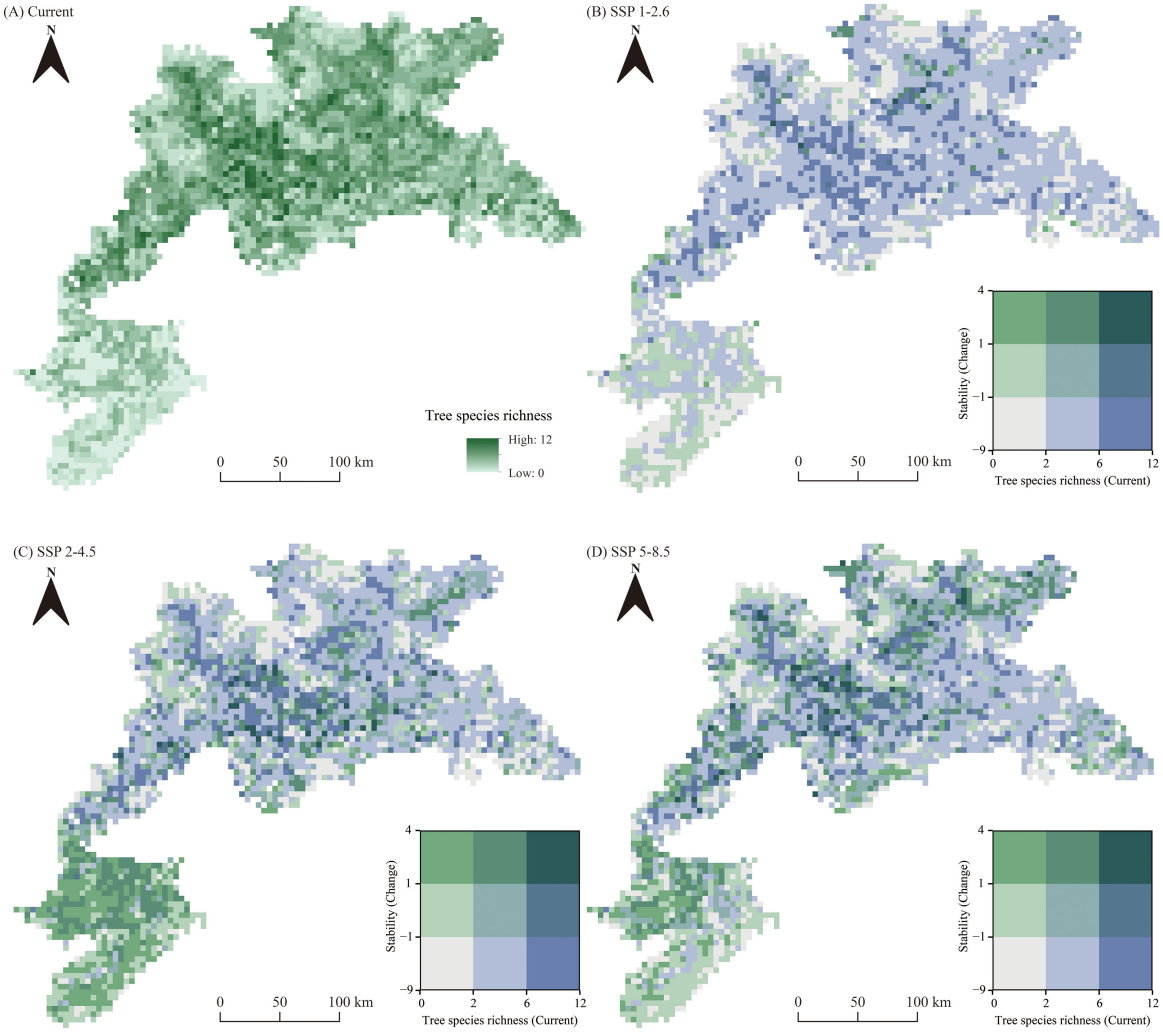
Bivariate spatial mapping of current tree species richness and projected net changes under future climate scenarios.

Based on the bivariate matrix integrating current species richness and net richness change (ΔS), three climate-trajectory zones were identified under each SSP scenario (**Figure 8**; **Table 4**). The high-risk degradation zone dominated under all scenarios but varied greatly in extent. Under SSP1-2.6 it covered 85.2% of the study area, far exceeding the climatically stable (13.2%) and gaining (1.6%) zones. Under SSP2-4.5 and SSP5-8.5 the degradation zone was much smaller (51.0% and 54.0%), while the stable zone (33.1% and 27.4%) and especially the gaining zone (15.9% and 18.7%) expanded substantially relative to SSP1-2.6. This exceptionally large degradation zone under SSP1-2.6 parallels the pronounced contraction of highly suitable habitat and the large centroid shifts observed under the same scenario.

**Table 4:** Area proportions of the three climate trajectory zones under different future climate scenarios.

climate trajectory zones	SSP1-2.6 (%)	SSP2-4.5 (%)	SSP5-8.5 (%)
High-risk degradation zone	85.2	51.0	54.0
Climatically stable zone	13.2	33.1	27.4
Climatically gaining zone	1.6	15.9	18.7

Zones are defined by net richness change (ΔS): degradation (ΔS ≤ −1), stable (ΔS = 0), and gaining (ΔS ≥ +1). Values are the percentage of the total study area; columns sum to 100%.

Cross-classifying current richness with climate trajectory revealed marked spatial heterogeneity (**Figure 8**; **Supplementary Table 3**). The high-richness degradation zone (high richness × ΔS ≤ −1) covered 11.0%, 6.5%, and 8.1% of the study area under SSP1-2.6, SSP2-4.5, and SSP5-8.5, respectively. These are areas that currently support the most suitable species yet face substantial climate-driven loss. By contrast, the high-richness stable zone (high richness × ΔS = 0), representing strong climate buffering, was far smaller (0.7%, 4.0%, and 2.7%) and was concentrated in the core limestone mountains of Yingde City and Yangshan County. The low-richness stable zone (low richness × ΔS = 0) was more extensive (11.0%, 16.0%, and 12.9%), occurring mainly along the western and eastern margins, where current richness is inherently low. Gaining areas spanned all richness levels and were most extensive under SSP5-8.5 (18.7% overall).

Under the current climate, species richness across the 12 dominant species was strongly heterogeneous in space (**Figure 8**). High-richness areas (7–12 suitable species; 11.9% of the region) formed a contiguous core in the limestone mountains of northern Guangdong, concentrated in Yingde City, Yangshan County, and Huaiji County. Fengkai, Renhua, and Wengyuan counties also contributed notably. By contrast, the southwestern margins (e.g., Yunfu and Luoding), where high-richness areas were essentially absent, and the eastern margins supported only low-to-moderate richness, with the low-richness class alone covering 34.9% of the region. High-richness degradation was most severe under SSP1-2.6, extending into the marginal high-richness zones, whereas climate-buffered stable refugia were minimal under this scenario; under SSP2-4.5 and SSP5-8.5, the stable zone within the northern mountainous core expanded. These marginal high-richness areas are currently suitable but face a high risk of species loss, most acutely under SSP1-2.6.

## 4 Discussion

While previous SDM studies in karst regions have predominantly focused on single-species assessments or broad-scale niche characterizations (Radbouchoom et al., 2025; Zhang et al., 2026), this study integrates multi-species modeling with bivariate spatial analysis to simultaneously address species level climate vulnerability and community level afforestation risk, offering a more operationally relevant framework for climate adaptive restoration planning. This study systematically quantifies the potential distribution patterns of 12 dominant tree species in the northern Guangdong limestone region and reveals the spatiotemporal dynamics of their suitable habitats under future climate scenarios, establishing a scientific foundation for climate adaptive afforestation planning and ecological restoration in fragile karst landscapes. The findings demonstrate that precipitation availability is the primary climatic constraint on species distributions in this region, that climate-driven habitat responses exhibit pronounced interspecific heterogeneity despite modest changes in total suitable area, and that areas of high current species richness do not necessarily correspond to the most resilient afforestation targets.

### 4.1 Reliability of the MaxEnt model

Although ensemble modeling is frequently advocated for SDM applications, a single-algorithm MaxEnt model can produce distribution maps of accuracy comparable to ensembles at substantially lower computational cost (Kaky et al., 2020; Schwarz et al., 2025). MaxEnt’s default parameters were tuned to reproduce realized distributions, not to transfer across time or space (Phillips and Dudík, 2008). We therefore optimized the regularization multiplier and feature classes, selecting the combination with ΔAICc = 0. This favored higher regularization values, which penalized model complexity and reduced overfitting, thereby improving transferability to novel future climates (Ahmadi et al., 2023; Radosavljevic et al., 2014; Valavi et al., 2022). Given the 12 target species and the demands of multi-scenario projection, this optimized single-algorithm approach was a pragmatic and defensible choice.

We nonetheless acknowledge that a single algorithm cannot capture the between-model variability an ensemble would quantify (Araújo and New, 2007). This uncertainty is expected to be greatest under high-emission scenarios, where projections extrapolate furthest beyond the sampled environmental space (Brun et al., 2020). Our SSP5-8.5 projections should accordingly be interpreted as the response of one well-calibrated model rather than a multi-algorithm consensus.

To translate these full-range predictions to the study region, we clipped them with a limestone geological mask for northern Guangdong. Each model was calibrated across the species’ full Chinese range and then clipped to the limestone region. In the spirit of nested SDMs (Guisan et al., 2025), this broad-to-local strategy samples the complete realized climatic niche rather than a geographically truncated subset (Anselmetto et al., 2025; Pang et al., 2022). It also restricts the final inference to ecologically meaningful boundaries, clarifying climate–distribution relationships at a region-relevant scale (Araújo and Pearson, 2005; Chauvier et al., 2022; Pearson and Dawson, 2003). The broad calibration extent is therefore expected to mitigate, rather than fully eliminate, niche truncation in the regional projections.

### 4.2 The key variables affecting the potential distribution of the 12 dominant tree species

Across the 12 species, precipitation was the dominant distributional driver, particularly annual precipitation (BIO12) and precipitation of the driest month (BIO14). Temperature acted as a secondary control, and in one species soil texture was the leading control. Two precipitation-governed species illustrate contrasting response forms that imply different moisture controls. One species exhibited a saturating growth response, while the other showed an intermediate optimal value. *C. mollissima* showed a saturating dependence on precipitation of the driest month (BIO14), with suitability increasing as dry-season rainfall rose. This accords with research that, although *Castanea* are heliophytic trees broadly adaptable to mountainous terrain, their tolerance of aridity is strictly time-limited (Braga et al., 2023). In karst terrain, where shallow soils over porous bedrock retain little rainfall (Yao et al., 2023), dry-season water availability may therefore become the decisive constraint, explaining why driest-month rather than annual precipitation dominated. *Z. insignis*, by contrast, responded to precipitation of the wettest quarter (BIO16) with an intermediate optimum rather than a saturating gain, indicating that it is favored by a moderate wet-season regime. Its drought-avoidance physiology (Liu et al., 2026a) constrains performance where wet-season input is low. Suitability also falls at the wettest extreme, defining a narrow optimum consistent with its restricted, fragmented habitat (**Table 3**). A second assemblage (*T. sinensis*, *C. sinensis*, and *M. azedarach*) was governed chiefly by annual precipitation (BIO12), with the mean temperature of the warmest quarter (BIO10) as a secondary thermal control. Modelled suitability for all three approached zero below approximately 25 °C and rose toward warmer conditions, implying a requirement for warm-season heat accumulation rather than tolerance of winter cold. For *T. sinensis*, this inference is independently corroborated by provenance-level germination trials, in which germination was optimal near 25 °C (Yousheng and Sziklai, 1985). That value matches the threshold below which modelled suitability collapsed. Among the three, thermal control was strongest in *C. sinensis*, for which BIO10 also carried the highest permutation importance. *L. communis* showed a dual control. Annual precipitation (BIO12) and driest-month precipitation (BIO14) indicate sensitivity to both total and dry-season water supply. Minimum temperature of the coldest month (BIO6) carried a permutation importance well above its percent contribution, indicating that the model relied substantially on winter cold to separate suitable from unsuitable sites. As an evergreen broadleaved species of the Lauraceae, *L. communis* retains its foliage through winter, so low winter temperatures may constrain its photosynthetic function and overwintering (Oquist and Huner, 2003). *B. minus* was distinctive in being led not by climate but by an edaphic factor. Its modelled preference for coarse, well-drained substrates is ecologically coherent in karst terrain, where shallow soils over fractured bedrock drain rapidly (Yao et al., 2023). This provides a clear basis for the unusually high contribution of soil texture in this species.

Some findings diverge from earlier single-region studies, and these divergences are most parsimoniously attributed to differences in regional environmental context rather than to the modeling itself. For *C. mollissima*, Dai et al. (2023) identified annual precipitation (BIO12) as the principal driver, whereas driest-month precipitation (BIO14) dominated here. This shift is consistent with the persistent dry-season water deficit of karst terrain, where dry-month moisture rather than annual total becomes the operative constraint. Similarly, Xiao et al. (2022b) reported temperature as the leading control on *C. axillaris*, whereas in our study the species was precipitation-governed. This plausibly reflects the warm, humid setting of the South China limestone region, where thermal conditions are rarely limiting and moisture supply instead becomes primary. In both cases, the dominant driver for a single species is conditional on its regional environmental context.

Beyond these individual controls, two broader patterns emerge. First, non-climatic predictors were far from negligible. In contrast to settings where elevation dominates the non-climatic signal through its effect on microclimate (Bátori et al., 2017), here the most influential non-climatic factor was soil texture, and only for *B. minus*. Although soil contributed little for most species, its decisive role there substantiates our inclusion of edaphic predictors and shows that soil data can materially improve predictions in heterogeneous karst terrain (Buri et al., 2017; Ni and Vellend, 2024). Second, the drivers trace a gradient from climatic generalists to specialists. Broadly tolerant species such as *C. sinensis* and *M. azedarach* occupied the largest highly suitable areas, whereas species hinging on a single narrow optimum (*Z. insignis*, *S. wightianum*, and *B. minus*) occupied the smallest, most fragmented habitats (**Table 3**). Because narrow environmental tolerance is generally associated with restricted range size and heightened vulnerability to environmental change (Slatyer et al., 2013), these limestone specialists warrant particular attention in climate-adaptive afforestation. Finally, the physiological mechanisms invoked above are inferred from the modelled environmental responses and the published ecophysiology of these or related taxa, not from direct measurement here. They are therefore advanced as plausible hypotheses for targeted ecophysiological and common-garden testing rather than as confirmed causal relationships.

### 4.3 Impact of climate change on the suitable habitats of the 12 dominant limestone tree species

Understanding how potential distributions shift under different climate scenarios is essential for assessing climate-change impacts and formulating adaptive forest-management strategies. In the northern Guangdong limestone region, the suitable habitat of the dominant tree species responded to future climate in a strongly scenario-dependent manner rather than contracting uniformly with rising emissions. Under SSP1-2.6, both total and highly suitable habitat declined sharply for most species. Under SSP2-4.5 and SSP5-8.5, total suitable area was largely restored for most species, but highly suitable habitat diverged. It expanded markedly in a few species, particularly for *S. wightianum*, the only species to gain highly suitable habitat under all three scenarios, while continuing to decline in others, including *C. sinensis*, *C. mollissima*, *D. myricoides*. This suggests that under the higher-emission scenarios, a stable or even expanding total suitable area can mask pronounced contraction of highly suitable habitat. This occurs in *C. sinensis* and *T. sinensis*, whose total extent changed little while their highly suitable core shrank. This decoupling of habitat quality from habitat extent has been reported for other subtropical Chinese trees, where the contraction of highly suitable habitat under more severe scenarios was interpreted as a deterioration of habitat quality rather than of area (Ren et al., 2025). The most severe contraction has been reported under intermediate rather than the highest-emission scenarios in some species (Xu et al., 2025), while others expand under low-emission pathways yet lose highly suitable habitat under high-emission ones (Ren et al., 2025). *T. sinensis* exemplifies the resulting core risk: under SSP5-8.5 its total suitable area was essentially unchanged, yet its highly suitable habitat contracted. This divergence would be missed if habitat change were judged by total extent alone. Together these indicate that the direction of the scenario response is region- and species-specific.

Rather than the coherent poleward tracking commonly reported under warming (Parmesan and Yohe, 2003; Chen et al., 2011), the centroid shifts were directionally heterogeneous and dominated, where consistent at all, by south-westward movement. This pattern may reflect tracking of an internal moisture or terrain gradient rather than a latitudinal thermal gradient. Such behavior is consistent with distributions governed primarily by moisture rather than temperature, for which warming imposes no straightforward poleward displacement. The limited centroid displacements not only reflect more than the modest latitudinal extent of the study area but also reveal the ecological reality of range tracking in fragmented karst. The discontinuous limestone matrix offers few continuous corridors of suitable habitat, so even where the climate envelope shifts, dispersal-limited species may be unable to follow it. This mismatch is widely recognized as climate-tracking lag (Lenoir et al., 2020; Bertrand et al., 2011). The constraint is mirrored in the directional structure of the migrations. Species lacking any stable bearing may have no clear climatic escape route, reversing direction as the suitable envelope fragments and re-forms among scenarios. By contrast, the rare species combining a consistent bearing with sustained habitat gain, exemplified by *S. wightianum*, are those for which a coherent tracking pathway may remain available. Realized redistribution will therefore depend not on the climate trajectory alone but on its coupling with each species’ dispersal capacity and the connectivity of the karst landscape.

Taken together, these patterns indicate that the principal climate-change threat to the dominant tree species of this region lies in the erosion of habitat quality rather than the loss of habitat extent. Total suitable area was broadly buffered under the higher-emission scenarios, yet highly suitable habitat contracted for most species, most severely under SSP1-2.6. Because the models resolve macro-scale climatic suitability but not local microclimates, dispersal, anthropogenic disturbance, or land use, they delineate the climatic potential within which these species can persist and be restored under future climates, rather than the precise extent to which they will do so. Moreover, because the distributions of these species are moisture-limited, and CMIP6 precipitation projections over South China carry markedly lower model skill than temperature (Peng et al., 2025), the SSP1-2.6 results are best interpreted as conditional on the regional precipitation projection of the single climate model employed (BCC-CSM2-MR). We therefore treat them with appropriate caution and note that multi-model ensembles would help establish their robustness, a limitation of the present single-GCM design.

### 4.4 Implications to researchers and policymakers

Quantifying the climate-driven spatial dynamics of these dominant tree species provides a scientific basis for regional restoration planning and forestry policy, and identifies moisture availability as the primary climatic constraint on their distributions (Chowdhary et al., 2025, 2026). On this basis, and treating the anomalous SSP1-2.6 projections with caution, species differed in climatic security under the higher-emission scenarios (SSP2-4.5 and SSP5-8.5). *M. azedarach* and *C. axillaris* retained large and broadly stable highly suitable areas, while *T. rotundifolia* and *S. wightianum* expanded; together these represent the more climatically secure options for near-term afforestation, though the gains of *T. rotundifolia* and especially *S. wightianum* proceed from small baselines and should be judged by absolute, not only relative, area. By contrast, *C. sinensis*, *C. mollissima*, *T. sinensis*, *D. myricoides*, *L. communis*, and *Z. insignis* showed persistent contraction of highly suitable habitat across scenarios. For these species, we recommend strengthened long-term monitoring of distribution dynamics together with *in-situ* conservation of viable populations within the climatically more stable core (Xu et al., 2021), complemented by *ex-situ* germplasm conservation as insurance against *in-situ* loss (Godefroid et al., 2010); the storage method should be matched to each species’ seed behavior, since recalcitrant-seeded taxa cannot be banked conventionally and require living collections or cryopreservation (Fernández et al., 2023). Given the fragmented limestone matrix and the limited habitat connectivity inferred above, adaptive management should instead favor *in-situ* protection and, where reallocation is warranted, redistribute planting effort toward the stable core rather than rely on long-distance translocation.

At the landscape scale, the bivariate framework coupling current richness with net richness change identifies candidate priority zones rather than definitive targets. High-richness areas conventionally favored for afforestation coincided with the greatest degradation risk along their marginal belts (e.g., Fengkai, Renhua, Wengyuan), whereas the high-richness core of the Yingde, Yangshan, and Huaiji limestone mountains retained net richness stability and emerged as comparatively more resilient candidates. These same areas are where the highly suitable habitats of the greatest number of species overlap, marking them as multi-species climate refugia that warrant spatial priority in regional conservation and afforestation planning (Bátori et al., 2021). This screen rests on climate suitability and species richness alone. Operational site selection must additionally weigh soil suitability, dispersal and habitat connectivity, biotic interactions, land use, and socioeconomic constraints, none of which the model resolves. It must also allow for the tendency of overlay-based suitability to overestimate plantable area at the working resolution. The framework therefore informs relative prioritization among sites rather than prescribing absolute choices (Yu et al., 2022, 2024).

### 4.5 Limitations and future outlooks

Despite the strong predictive performance of the optimized MaxEnt models, projecting climate change impacts on species distributions over extended periods inevitably involves uncertainties and limitations. First, the models did not explicitly incorporate biological interactions such as interspecific competition and mutualism, nor anthropogenic pressures including land use change and logging (Wisz et al., 2013). Within authentic and highly heterogeneous karst habitats, the convergence of these abiotic and biotic factors may render the actual distribution of species substantially more restricted than the model predictions. Furthermore, the projection of future scenarios in this study treated soil and topographical attributes as static variables (Lehmann and Kleber, 2015). However, within limestone mountainous regions, the intensification of climate warming and extreme precipitation events may gradually alter the hydrological connectivity and physicochemical properties of soils. Consequently, this static assumption partially overlooks the dynamic coupling effects between climate and soil. Finally, the models implicitly postulate a capacity for unlimited migration and dispersal among species. In reality, however, the endemic species of the northern Guangdong limestone region frequently encounter habitat fragmentation and geographical dispersal barriers induced by topographic dissection. Therefore, the actual spatial evolution of suitable habitats may lag behind the theoretical predictions. Future research should incorporate dynamic environmental variables, multispecies interaction networks, and explicit dispersal constraints to improve the ecological realism of long-term spatial predictions.

## 5 Conclusion

Using optimized MaxEnt models under three future climate scenarios, this study quantified the potential suitable distributions of 12 dominant tree species across the northern Guangdong limestone region. It further resolved how their habitats shift in space and time as the climate changes. Precipitation-related variables were the primary drivers, led by annual precipitation (BIO12) and precipitation of the driest month (BIO14), whereas mean temperature of the warmest quarter (BIO10) was the principal temperature constraint. Individual species nonetheless showed strong idiosyncratic controls, most notably wettest-quarter precipitation (BIO16) for *Z. insignis* and topsoil texture (T_USDA_TEX) for *B. minus*. Under future warming, the change in total suitable area stayed modest for every species, but the change in highly suitable habitat was far more pronounced. This confirms that the extent of highly suitable habitat is a more sensitive indicator of climate vulnerability than total suitable area. Species responses were heterogeneous and strongly scenario dependent. Highly suitable habitat contracted for 11 of the 12 species under SSP1-2.6, and only *S. wightianum* gained across all three scenarios. *T. rotundifolia* expanded under SSP2-4.5 and SSP5-8.5 yet contracted under SSP1-2.6. Notably, the low-emission SSP1-2.6 scenario produced the most severe and most spatially erratic habitat degradation. This indicates that, in this region, lower-emission pathways do not necessarily translate into lower restoration risk. Centroid migration distances did not show dominant directional trend across the assemblage, likely reflecting the narrow latitudinal extent of the study area. The bivariate spatial analysis showed that areas of high current species richness face disproportionately high climate-driven degradation risk. Within that high-richness core, the climatically stable zones of the limestone mountains in Yingde City and Yangshan County represent the most resilient targets for potential priority areas for climate-resilient afforestation planning. Together, these findings provide a spatially explicit, climate-informed framework for tree species selection and afforestation site prioritization in the karst regions of southern China. These projections are based on climatic suitability and do not incorporate dispersal limitations, biotic interactions, or land-use change, so it is recommended that they be validated in the field and assessed for uncertainty before being applied to planning. The framework has direct relevance for regional ecological restoration planning and long-term forest resource management under a changing climate.

## Data Availability

The original contributions presented in the study are included in the article/**Supplementary Material**. Further inquiries can be directed to the corresponding author.
