# Supplementary material for: Predicting potential distributions of dominant species and assessing future afforestation risks in Guangdong limestone regions under climate change
Source: Front Plant Sci. 2026 Jul 22;17:1870529. doi: 10.3389/fpls.2026.1870529 (PMC13437973; doi:10.3389/fpls.2026.1870529)
Supplement: Supplementary file 1 [file DataSheet1.docx]

Supplementary Material

# 1 Supplementary Tables

**Supplementary Tables 1.** Environment variables

| type | Variables | Description | Unit |
| --- | --- | --- | --- |
| bioclimatic | Bio1 | Annual mean temperature | °C |
|  | Bio2 | Mean diurnal range (Mean of monthly) | °C |
|  | Bio3 | Isothermality (Bio2/Bio7) (× 100) |  |
|  | Bio4 | Standard deviation of temperature seasonality |  |
|  | Bio5 | Max temperature of warmest month | °C |
|  | Bio6 | Min temperature of coldest month | °C |
|  | Bio7 | Temperature annual range (Bio5-Bio6) | °C |
|  | Bio8 | Mean temperature of wettest quarter | °C |
|  | Bio9 | Mean temperature of driest quarter | °C |
|  | Bio10 | Mean temperature of warmest quarter | °C |
|  | Bio11 | Mean temperature of coldest quarter | °C |
|  | Bio12 | Annual precipitation | mm |
|  | Bio13 | Precipitation of wettest month | mm |
|  | Bio14 | Precipitation of driest month | mm |
|  | Bio15 | Variation of precipitation seasonality |  |
|  | Bio16 | Precipitation of wettest quarter | mm |
|  | Bio17 | Precipitation of driest quarter | mm |
|  | Bio18 | Precipitation of warmest quarter | mm |
|  | Bio19 | Precipitation of coldest quarter | mm |
| soil | T_GRAVEL | gravel volume percentage | % |
|  | T_SAND | sand content | % |
|  | T_SILT | silt content | % |
|  | T_CLAY | clay content | % |
|  | T_USDA_TEX | USDA soil texture classification |  |
|  | T_OC | organic carbon content | % |
|  | T_PH_H2O | pH |  |
|  | T_CACO_3_ | carbonate or lime content | % |
| terrain | Elev | elevation | m |
|  | Slo | slope | ° |
|  | Asp | aspect |  |

**Supplementary Tables 2.** Changes in the center of gravity of the optimal distribution areas for 12 dominant tree species under future climate scenarios compared to current conditions

| Species | Scenario | Longitude (°E) | Latitude (°N) | Direction | Distance (km) |
| --- | --- | --- | --- | --- | --- |
| *Zenia insignis* | Current | 113.253 | 24.239 | — | — |
|  | SSP126 | 112.918 | 24.453 | NW | 44.2 |
|  | SSP245 | 112.89 | 24.098 | SW | 43.2 |
|  | SSP585 | 113.094 | 24.28 | NW | 18.3 |
| *Quercus glauca* | Current | 113.533 | 24.746 | — | — |
|  | SSP126 | 113.842 | 24.71 | SE | 34.6 |
|  | SSP245 | 113.766 | 24.863 | NE | 28.9 |
|  | SSP585 | 113.519 | 24.512 | SW | 26.0 |
| *Choerospondias axillaris* | Current | 113.288 | 24.315 | — | — |
|  | SSP126 | 113.046 | 24.27 | SW | 27.3 |
|  | SSP245 | 113.28 | 24.284 | SW | 3.6 |
|  | SSP585 | 113.196 | 24.257 | SW | 12.1 |
| *Triadica rotundifolia* | Current | 113.15 | 24.214 | — | — |
|  | SSP126 | 112.978 | 24.658 | NW | 52.9 |
|  | SSP245 | 112.958 | 24.376 | NW | 27.9 |
|  | SSP585 | 112.959 | 24.056 | SW | 27.6 |
| *Lindera communis* | Current | 113.389 | 24.407 | — | — |
|  | SSP126 | 113.089 | 24.816 | NW | 56.3 |
|  | SSP245 | 113.36 | 24.417 | NW | 3.3 |
|  | SSP585 | 113.426 | 24.358 | SE | 6.9 |
| *Toona sinensis* | Current | 112.907 | 24.153 | — | — |
|  | SSP126 | 113.232 | 24.804 | NE | 80.8 |
|  | SSP245 | 112.885 | 24.232 | NW | 9 |
|  | SSP585 | 113 | 24.203 | NE | 11.7 |
| *Sinosideroxylon wightianum* | Current | 112.303 | 23.343 | — | — |
|  | SSP126 | 112.417 | 23.462 | NE | 18.3 |
|  | SSP245 | 112.323 | 23.423 | NE | 9.1 |
|  | SSP585 | 112.387 | 23.459 | NE | 15.8 |
| *Celtis sinensis* | Current | 113.132 | 24.265 | — | — |
|  | SSP126 | 113.267 | 24.516 | NE | 31.7 |
|  | SSP245 | 112.87 | 24.103 | SW | 34.1 |
|  | SSP585 | 112.947 | 24.061 | SW | 30.5 |
| *Castanea mollissima* | Current | 113.261 | 24.355 | — | — |
|  | SSP126 | 113.193 | 24.422 | NW | 10.5 |
|  | SSP245 | 113.281 | 24.5 | NE | 16.2 |
|  | SSP585 | 113.132 | 24.288 | SW | 16 |
| *Distylium myricoides* | Current | 113.366 | 24.417 | — | — |
|  | SSP126 | 113.255 | 24.567 | NW | 20.7 |
|  | SSP245 | 113.317 | 24.448 | NW | 6.5 |
|  | SSP585 | 113.401 | 24.648 | NE | 25.9 |
| *Boniodendron minus* | Current | 112.64 | 23.673 | — | — |
|  | SSP126 | 113.007 | 24.406 | NE | 90.9 |
|  | SSP245 | 112.624 | 23.727 | NW | 6.3 |
|  | SSP585 | 112.622 | 23.656 | SW | 2.8 |
| *Melia azedarach* | Current | 113.237 | 24.378 | — | — |
|  | SSP126 | 113.309 | 24.518 | NE | 17.5 |
|  | SSP245 | 113.15 | 24.287 | SW | 14 |
|  | SSP585 | 112.932 | 23.996 | SW | 54.2 |

**Supplementary Tables 3.** Cross-classification of current species richness and projected climate trajectory (ΔS), expressed as the percentage of the total study area under each future scenario. Current species richness was classified as low (0–2 species), moderate (3–6 species), or high (7–12 species). The climate trajectory was defined by the projected change in species richness (ΔS = future − current richness): degradation (ΔS ≤ −1), stable (ΔS = 0), and gaining (ΔS ≥ +1). Values represent the percentage of the total study area falling in each richness × trajectory combination under each scenario; columns sum to 100%.

| Richness Level | Climate Trajectory Zone | SSP126 (%) | SSP245 (%) | SSP585 (%) |
| --- | --- | --- | --- | --- |
| High species richness (7–12 species) | degradation zone | 11 | 6.5 | 8.1 |
| High species richness (7–12 species) | Stable Zone | 0.7 | 4 | 2.7 |
| High species richness (7–12 species) | Gain Zone | 0.2 | 1.4 | 1.1 |
| Moderate species richness (3–6 species) | degradation zone | 50.9 | 31.6 | 32.9 |
| Moderate species richness (3–6 species) | Stable Zone | 1.6 | 13.1 | 11.8 |
| Moderate species richness (3–6 species) | Gain Zone | 0.7 | 8.5 | 8.5 |
| Low species richness (0–2 species) | degradation zone | 23.2 | 12.9 | 13 |
| Low species richness (0–2 species) | Stable Zone | 11 | 16 | 12.9 |
| Low species richness (0–2 species) | Gain Zone | 0.7 | 6 | 9.1 |

# 3 Supplementary Tables

**Supplementary** Figure 1 Response curves of two species‑specific leading variables, derived from the optimized MaxEnt models and averaged across 10 bootstrap replicates. (A) Topsoil texture (T_USDA_TEX) for *Boniodendron minus*; (B) precipitation of the wettest quarter (BIO16) for *Zenia insignis*. The y‑axis is the logistic habitat suitability index.

**Supplementary Figure 2** Potential geographical distribution of 12 dominant tree species in the northern Guangdong limestone region under the SSP1-2.6 climate scenario.

**Supplementary Figure 3** Potential geographical distribution of 12 dominant tree species in the northern Guangdong limestone region under the SSP2-4.5 climate scenario.

**Supplementary Figure 4** Potential geographical distribution of 12 dominant tree species in the northern Guangdong limestone region under the SSP5-8.5 climate scenario.
